# The roles of healthcare professionals in diabetes care: a qualitative study in Norwegian general practice

**DOI:** 10.1080/02813432.2020.1714145

**Published:** 2020-01-21

**Authors:** Monica Sørensen, Karen Synne Groven, Bjørn Gjelsvik, Kari Almendingen, Lisa Garnweidner-Holme

**Affiliations:** a Faculty of Health Sciences, Department of Nursing and Health Promotion, OsloMet University, Oslo, Norway;; b Faculty of Health Sciences, Department of Physiotherapy, OsloMet University, Oslo, Norway;; c Department of General Practice, Institute for Health and Society, University of Oslo, Oslo, Norway;; d Faculty of Health Sciences, Department of Nursing and Health Promotion, OsloMet University, Oslo, Norway

**Keywords:** General practice, multi-professional collaboration, diabetes mellitus, nurse, medical secretary

## Abstract

**Objective:** To explore the experiences of general practitioners (GPs), nurses and medical secretaries in providing multi-professional diabetes care and their perceptions of professional roles.

**Design, setting and subjects:** Semi-structured interviews were conducted with six GPs, three nurses and two medical secretaries from five purposively sampled diabetes teams. Interviews were analysed thematically.

**Main outcome measures:** Healthcare professionals’ (HCPs’) experiences of multi-professional diabetes care in general practice.

**Results:** The involvement of nurses and medical secretaries (collaborating health care professionals) was mainly motivated by GPs’ time pressure and their perception of diabetes care as easy to standardize. GPs reported that diabetes care had become more structured and continuous after the involvement of collaborating health care professionals (cHCPs). cHCPs defined their role differently from GPs, emphasizing that their approach included acknowledging patients’ need for diabetes education, listening to their stories and meeting their need for emotional support. GPs appeared less involved in patients’ emotional concerns and more focused on the biomedical aspects of illness. There was little emphasis on teamwork among GPs and cHCPs, and none of the practices used care plans to involve patients in decisions or unify treatment among professionals. Participants stated that institutional structures including a discriminatory remuneration system, lack of role descriptions and missing procedures for collaborative approaches were an obstacle to MPC.

**Conclusions:** cHCPs worked independently under delegated leadership of the GPs. Although cHCPs had a complementary role, HCPs in general practice may not take full advantage of the potential of sharing patient responsibility and learning with, from and about each other. Contextual barriers for team-based care approaches should be addressed in future research.

**KEY POINTS**
- It has been suggested that multi-professional approaches improve quality of care in people with long-term conditions.
- In this study, nurses and medical secretaries perceived to have a complementary role to general practitioners (GPs) in diabetes care, focusing on patient education, building trusting relationships and providing patients with emotional support.
- As multi-professional collaboration was minimal, GPs, nurses and medical secretaries in the included practices may not take full advantage of the potential of sharing care responsibility and learning with, from and about each other.

## Introduction

Globally, more people live with one or more long-term conditions (LTCs), accentuating the demand for complex primary care services [1]. In general practice, it is proposed that bringing together healthcare professionals (HCPs) with different perspectives, knowledge and skills improves patients' experience of care and enhances the working life of HCPs [2–6]. Workforce transformation based on new models of care and skill-mix change may also increase care efficiency and efficacy [7]. For example, estimates from the USA show that almost 50% of care for patients with LTCs and up to 80% of preventive care could be performed by non-physician members of the general practice team [8–10].

Multi-professional collaboration (MPC) in health care is the process by which interdependent professionals structure a collective action towards patients’ care needs [11]. Despite growing recognition of the importance of collaborative approaches mandated in policy reforms in Norway and globally [12–14], healthcare institutions struggle to define and achieve new forms of collaborative practice [15,16]. MPC in healthcare lacks clear conceptualisations [17,18]. There is limited empirical evidence to guide practice transformation in developing new standards of care, in which knowledge, decisions and responsibility are shared [19,20]. A systematic review, exploring team-building interventions in non-acute healthcare settings, found little evidence to describe the determinants of professional interaction [21].

The average general practice in Norway has 3.6 general practitioners (GPs), providing care for 1,106 patients per GP [22,23]. About 95% of general practices are owned by GPs on contract with the municipality, financed by capitation, fee-for-service and patient co-payment. In many countries, the shift from task delegation to team-based care has followed the introduction of new reimbursement schemes, such as pay for performance, capitation and direct subsidies to employ and train nurses [24,25]. In Norway, only physician-led care is eligible for reimbursement in general practice, whilst collaborating healthcare professionals (cHCPs), such as nurses, medical secretaries and dietitians lack the authority to bill for their services independently and are directly employed by practices. Hence, compared to some other countries [24,26], multi-professional team-based care is not common in Norwegian primary care.

Diabetes mellitus is a complex disease, and Norwegian guidelines for treatment of diabetes emphasise the demand for patients and carers to attend to multiple psychological, behavioural and environmental factors and their interactions [27]. A meta-regression analysis summarizing the most effective quality improvement interventions in T2DM found that expanding professional roles, team-based approaches and case management were the most effective in reducing HbA1c [28]. Some Norwegian general practises have re-organized diabetes care to a more collaborative approach, involving nurses or medical secretaries. However, little is known about the experience of MPC and the roles and care approaches adopted by various professionals in new forms of collaborative constellations [24]. Our recent scoping review could not identify any publications reporting from MPC in Norwegian general practice [29]. Thus, scientific studies have not investigated the experiences of the few multi-professional teams operating in Norwegian general practice. In a global perspective, more studies about factors influencing the collaboration of HCPs in diabetes care in general practice are needed. Given this gap in knowledge, we sought to explore the experience of GPs, nurses and medical secretaries in some of these practices.

We posed the following research question:

- How do GPs, nurses and medical secretaries experience their role and care approach in multi-professional diabetes care in general practice?

## Materials and methods

### Setting, recruitment and participant characteristics

This is a qualitative and exploratory study, drawing on interviews with six GPs, three nurses and two medical secretaries. Five general practices were purposively sampled, acknowledged by physician colleagues and the authors as providing multi-professional follow-up of patients with type 1 diabetes mellitus (T1DM) and type 2 diabetes mellitus (T2DM). All practices were privately owned and run by 4–7 GPs (see Table 1 for practice demographics). All but one interview was conducted face-to-face (the final interview was performed via phone for practical reasons (cHCP4)).

**Table 1.** Demographics of included practices.

Practice	Practice composition	Rural/Urban^a^
1	7 GPs, 0 nurses, 8 secretaries	Urban
2	5 GPs, 3 nurses, 2 secretaries	Rural
3	6 GPs, 1 nurse, 4 secretaries	Urban
4	4 GPs, 2 nurses, 5 secretaries	Urban
5	6 GPs, 5 nurses, 3 secretaries	Rural

^a^Rural: place with <20,000 inhabitants, Urban: city with >20,000 inhabitants.

One GP in each practice received written information describing the study’s purpose and aims. This included the following statement: “We wish to study how diabetes care is organized in your practice and how healthcare providers from different professional backgrounds collaborate in providing diabetes care”. In particular, we stated that we wanted to explore professional roles, care approaches and how patients were involved in care decisions. In this study, we use the term “multi-professional” to reflect the way in which team members worked. In contrast to *inter-professional* collaboration, which refers to collaboration involving the continuous sharing of information and decisions as well as a more team-based approach, the professionals included in this study worked mainly independently [30,31].

The contact GPs were responsible for scheduling interviews with HCPs in their own practice and distributing participant consent forms. The included practices had twelve years (range: 7–15 years) experience of working in a multi-professional setting with diabetes. All cHCPs were female and all but one GP were male. Two of the cHCPs were medical secretaries. Two of the three nurses were diabetes specialist nurses. Participant characteristics are summarized in Table 2 (these data were provided orally by the participants at the end of the interviews). To ensure participant confidentiality, numbers are given as mean/range.

**Table 2.** Characteristics of healthcare professionals included.

Interviewee	Age (mean/range)	Experience in years, (mean/range)	Patients per week or list size^a^
cHCP1 (medical secretary)	48 (43–54)	15 (10–25)	9
cHCP2 (diabetes specialist nurse)			7
cHCP3 (medical secretary)			3
cHCP4 (nurse)			30
cHCP5 (diabetes specialist nurse)			15
GP1	50 (31–69)	23 (3–42)	1,420
GP2			600
GP3			1,550
GP4			1,200
GP5			1,000
GP6			1,480

a The average number of patients per week is given for cHCPs. List size is given for GPs.

### Data collection

The semi-structured interview guide was theoretically underpinned by three of the core competencies for caring for patients with long-term conditions issued by the WHO and includes: 1) the skills of professionals in collaborating with each other and with patients, 2) the development of common treatment plans based on patient goals and 3) implementation strategies focusing on the needs, values, preferences and self-management skills of patients [32]. The interview guide can be found in Appendix I.

The applicability and time requirement of the interview guide were tested in a pilot interview. As this necessitated only minor adjustments (e.g. removing a question about inter-professional collaboration), it was included in our final analysis. Interviews were conducted individually in private consultation rooms at each general practice by MS and lasted 20–60 min. The phone interview lasted for 30 min. Following all interviews, descriptions of the interview context were immediately recorded by the interviewer.

### Data processing and analysis

All interviews were audiotaped, transcribed verbatim and analysed thematically using Braun and Clarke’s methodology [33]. Transcripts were read and re-read, and initial codes were developed by identification and grouping of meaningful units of text based on their relevance to our research question. Comprehensive extracts of text were then condensed, labelled with codes and collated under thematic headings. Citations were transcribed from Norwegian into English by the first author.

Two key professional competencies guided the data analysis: person-centred care (PCC) and professional partnering [32]. We used the term *person-centred care* (as compared to patient-centred care), concurring the importance of a holistic focus on the patient over time and independent of particular diseases. Moreover, PCC is concerned with patients’ experienced health problems, overall wellbeing and function in daily life [34,35]. In care settings, professional partnering involves the ability to communicate in a way that enables professional collaboration and partnering with patients.

To ensure theme and sub-theme consistency, coherence, and distinctiveness, we compared and contrasted similar codes and developed an initial coding tree. Each code was briefly described and checked against the original data using an iterative and reflexive process. We then described and interpreted the themes and sub-themes to explicate connections, contradictions and hidden meanings. The authors shared and reflected on a descriptive summary of preliminary themes and sub-themes in order to enhance the credibility of the findings. To validate the premise that themes and sub-themes were representative and remained directly linked to the statements of the participants, the researchers closely scrutinized the stages of data analysis and code assignment multiple times [36].

### Ethical considerations

At the outset of each interview, the interviewer reiterated the purpose and method of the study, participants' right to withdraw from the study at any time, emphasizing that data from the interview would be treated confidentially and confirming that personally identifiable information would be redacted in the transcripts. All participants (including the participant in the pilot interview) gave their informed consent for the interview to be audiotaped and transcribed.

## Results

The analysis of participants’ perception of multi-professional diabetes care revealed two main themes: *Complementary diabetes care* and *Role ambiguity.* In the following, we will elaborate on these two themes and corresponding sub-themes.

### Complementary diabetes care

#### Providing diabetes care in parallel

Typically, patients with diabetes were referred by their GPs to a designated nurse or medical secretary in the same practice, who performed diabetes controls independently. cHCPs had their own patient schedule with access to patient’s electronic medical record (EMR). They explained how they devoted a considerable amount of time searching the EMR to identify whether a patient had attended the clinic between diabetes controls and on reading record entries from previous appointments. The first consultation with a newly diagnosed patient was typically described by cHCPs as being comprehensive. It included retrieving the patient’s full medical history and detailed recordings of eating and physical activity habits. When asked, neither nurses nor medical secretaries confirmed that they applied this information systematically to improve care coordination. For example, none used this information to develop a shared care plan or to unify treatment goals with patients' preferences or among care providers.

GPs emphasized that adopting a multi-professional approach might lead to several positive effects. First, when patients were seen by the same nurse or medical secretary over a period of time, GPs noted that the consistency and continuity of diabetes care improved. Second, because cHCPs focused solely on diabetes, their care was perceived as being more predictable in terms of content and structure compared to the multifaceted GP-led care. Third, working collaboratively made GPs more aware of their own practice as they were responsible for the training of cHCPs. For example, several GPs admitted that their adherence to national guidelines and their inclination to stay up to date on research, technology and new medications had improved following task delegation of diabetes care to cHCPs. Finally, several GPs stated that the involvement of professions with different perspectives as compared to their medical point of view enhanced the overall understanding of patient complexity and needs. One GP explained the benefits of collaborating with a nurse who knew his patients well:

If one of our patients with diabetes struggles to achieve the HbA1c target, I usually go and talk with the nurse. She knows more about each patient’s life with diabetes and can easier detect where the shoe pinches. The alternative would be calling the endocrinologist, but he can only answer theoretically, not give any personal advice for this specific patient (GP3).

#### Diabetes as a case for multi-professional care

Participating GPs considered diabetes to be a favourable case for delegation of tasks because of easily standardized controls and because this group of patients often visited the general practice. Moreover, GPs affirmed that before entrusting partial responsibility of diabetes care to a nurse or medical secretary, their diabetes controls had been inconsistent, random and time-consuming. One of the GPs stated that his major motivation for involving the medical secretary in diabetes care was to ensure a more systematic approach to diabetes follow-up, thereby hopefully enhancing the quality of care:

Type 2 diabetes (T2DM) care was not well organized. With a hectic schedule and a high level of multimorbidity, diabetes was regularly forgotten. We lacked an effective system and the quality of care was too low. When had the patient’s feet been last checked, when had he last seen the ophthalmologist and the podiatrist and had I remembered to control his blood glucose levels? (GP1).

Both cHCPs and GPs used terms such as “hasty”, “unstructured”, “less available” and “too multifaceted” to describe GP’s diabetes consultations. Several GPs stated that their attitude could hamper a patient’s inclination to ask questions. One of the GPs compared patient involvement in his consultations to that of the medical secretary in order to demonstrate why collaboration improved the quality of care:

It’s easier to ask the secretary questions because she has more time than I do, and this allows the patient to talk without interruptions. There is often a tense atmosphere in my consultations. I’m always behind schedule, which does not pave the way for a lot of questions (GP1).

GPs’ motivation to involve cHCPs in the care of patients with other conditions than diabetes (examples given by the interviewer included mental illness, arthritis, asthma and cardiovascular disease) was not prioritized due to the demand for training and the maintenance of competence and skills. Also, several GPs emphasized the importance of not assigning complete responsibility for certain diseases to other professionals as they risked being unable to stay updated with good practice themselves.

Most GPs regarded difficult cases, including patients with multimorbidity or patients requiring continuous adjustment of glucose-lowering medications, to be unsuitable for referral to cHCPs. They explained this routine was to avoid unnecessary patient re-visits. One of the GPs argued his rationale of selecting certain patients for referral to the nurse and not others:

I don’t have any particular criteria for deciding which patients to refer to the nurse. I tend to mainly hang on to patients whose blood glucose is difficult to control and the intricate cases where I know the nurse will consult me anyway. A GP’s mind-set is practical and effective. I only refer patients that I know the nurse can handle herself without asking me about everything. Also, I believe she appreciates this independence. She takes notes in the EMR and I read through them as a quality control (GP5).

#### Increased focus on person-centred diabetes care

Participants stated that GPs consultations lasted 15–20 min, whereas cHCPs typically allocated 30 to 60 min for each consultation, adjusted to individual patient’s needs. Most cHCPs regarded successful patient cases as being the result of having a long-term perspective on treatment goals and sufficient time to get to know each other through conversations about everyday life issues. Giving patients time to adjust to new behaviours in a stepwise manner was perceived to diminish disease-related concerns and increase patients’ sense of self-management, as the following medical secretary explained:

I had a patient once who came back to me and said: “Actually, I’m glad I have type 2 diabetes. My quality of life has improved. I have quit smoking, I adhere to a regular exercise routine and I’m more aware of what I’m eating”. It’s funny, but he suddenly took command of his life (HCP1).

In general, cHCPs considered it important to reflect on how their care approach influenced patients’ feelings and motivation for self-management. For example, they strategically incorporated informal talk into their consultations as they felt this created a relaxed atmosphere in which patients could communicate more freely. One of the nurses stated that she worried that her patients regarded her consultations as examinations. She was particularly aware of not pushing or judging patients whose laboratory results were above the treatment goals. Another nurse emphasized how she was reluctant to provide patients with too much information. Instead, she tried to detect a patient’s readiness for change by encouraging participatory decision-making:

I don’t have all the answers, I can’t tell the patient to do this and do that. Rather, I can ask: What do you think? How can I help you reach your goals? (HCP5).

Further elaborating on how they focused on listening to patients and building trusting relationships, cHCPs emphasized the importance of remembering patients’ individual circumstances. Their narratives were drawn towards the communicative strategies they had adopted, specifically targeting diabetes self-management support (SMS). These techniques included motivational interviewing and guided self-determination, which cHCPs explained helped them connect with the person behind the disease and be sensible to the preferences and needs of individual patients. Conforming to the philosophical underpinnings of PCC, cHCPs emphasized the importance of being personally and sincerely engaged in their relationships with patients, as illustrated by this quote from one of the nurses:

All patients are unique. You must always consider whether someone is showing signs of resistance or information overload. Nobody benefits from setting goals that are too stringent. If I sense resistance, I always give the patient some more time. I want them [the patients] to feel that I am carrying some of the burden for them. I am very passionate about my work (HCP2).

In contrast, GPs appeared to be more concerned about the biomedical parameters and achieving targets for blood pressure, blood glucose and lipids. When asked explicitly, none of the GPs stated that they used any specific methods to involve and empower patients during consultations. Rather, they regarded patients’ superior need was information about their diseases and that the GP role entailed providing patients with oral and written advice, as this GP explained:

I prefer it if the patient reads about diabetes at home. There is a distinct limit in patients’ ability to understand what I’m saying during the short time we spend together” (GP4). When the same GP was asked whether he had a method for encouraging diabetes self-management, he replied: “No, I don’t have any specific method. It’s challenging when patients are unable to understand why it is important to change their behaviour (GP4).

### Role ambiguity

#### Different perceptions of competence required in diabetes care

On average, participating cHCPs had over 15 years of practice experience. Roles and responsibilities of cHCPs varied among the practices and originated in personal motivation and aspirations (e.g. one of the secretaries had T1DM herself), as well as the mind-set of the GPs. Preparing for their extended roles, medical secretaries had received one-on-one training from GPs at the practice and participated in several diabetes workshops and conferences. Thus, cHCPs’ competence largely depended on GPs’ priorities, GPs' propensity to remain updated about diabetes care and their willingness to share knowledge. For example, one of the GPs explained that the other GPs at the practice did not agree about the extent to which nurses should be involved in patient care:

Several of the other GPs do not agree with my own practice of delegating clinical tasks to our nurses. They’re not used to it. It’s a process and it starts with establishing trust and reassuring the nurses that you share the same philosophy of practice. It takes a lot of effort to convince physicians that you don’t have to be a doctor to do many of the things we are doing (GP6).

Similarly, there was disagreement among the GPs about the value of employing nurses in general practice. One GP, having more than 30 years of practice, regarded nurses as being over-qualified for working in general practice, whereas another GP emphasized that nurses covered more than 50% of the non-physician staff positions in his practice. The latter GP justified employing nurses by endorsing their ability to make independent and correct decisions in the reception, on the phone and in the laboratory. This practice had a clear policy of nurses maintaining their clinical integrity and not performing administrative tasks, which were entrusted to the medical secretaries.

All nurses emphasized that their competence played an important role in performing their daily tasks. When asked about the different roles between themselves and the medical secretaries, nurses emphasized their ability to make independent clinical judgements, thereby saving physicians’ time. One nurse used electrocardiography (ECG) controls as an example:

The secretaries might perform an ECG, but they cannot evaluate whether the patient should be seen by a physician immediately or whether they can go home (HCP5).

In contrast, the two medical secretaries did not recognize the need to employ nurses in general practice. Rather, they thought the competence of medical secretaries was superior to that of nurses because it is more targeted towards general practice and that secretaries could be trained in new roles.

Although disagreeing on roles, nurses and medical secretaries stated that being flexible was essential in order to manage diverse and unpredictable inquiries, often accompanied by staff shortages. Nurses were particularly aware of the additional cost they represented and felt obliged to increase the effectiveness and turnover of the practice, as captured in this statement from one of the nurses:

We must continuously evaluate how we can run this clinic more efficiently. I am able to perform several tasks simultaneously. Instead of waiting for a GP to come and see my patient, I can receive phone calls, take an ECG, remove stitches or assist the girls in the laboratory. We must consider the financial burden of employing nurses at the clinic, and justify the additional expense, as well as always consider what we can do to increase the flow of patients (HCP5).

## Discussion

This study explored perceptions of roles and care approaches of GPs, nurses and medical secretaries and brings important perspectives about factors influencing the collaboration of healthcare professionals in diabetes care in general practice. Our results indicate that cHCPs may complement medical care provided by GPs. By allocating more time than the GPs to each consultation and acting person-centred, cHCPs in our study sought to improve patients’ access to continuous and individualized diabetes care.

Studies from the UK, Germany and Denmark suggest that involving nurses in diabetes care is associated with improved quality of diabetes management and significant GP time saving with no adverse effects [37,38]. However, these studies do not provide insights into the ways in which nurses seek to increase care quality when working together with GPs. Interestingly, whilst GPs in our study described that the primary responsibility of cHCPs was to follow a standardized diabetes control, statements made by nurses and medical secretaries indicated that their focus also involved meeting patients’ psychological and emotional needs. Both nurses and medical secretaries stated that they attempted to communicate with patients using a conversational, personal and empowering style of interaction, whereas GPs characterized their own approach of clinical reasoning as being consultative and guided by test results. In this context, cHCPs seemed to supplement GP-led care. This finding aligns with previous research from primary care, reporting that nurse-led consultations are experienced by patients as more informal and friendlier than GP-led consultations [39,40].

PCC may improve patient’s knowledge, physical and psychological health, and ability to cope, and may lead to more appropriate clinical decisions [41]. The nursing profession has been referred to as “organizational glue” - a notion that has been linked to traditional gender roles. Indeed, women in healthcare are suggested to orient their attention to the needs of others, taking care of organisational needs, co-workers, and practical arrangements for patients and their families, seeking to manage functional gaps in the work place [42]. In this sense, women may naturally act in a more person-centred manner in general and the roles cHCPs in our study had adopted may therefore relate not only to their formal function, but also to inherent and traditional roles in being women.

Our results illustrate how nurses and medical secretaries worked independently under delegated leadership of their practice physicians, rather than attaining to a team-based approach. None of the practices organized joint meetings in which all professionals involved in diabetes care discussed professional roles, agreed on a common method of patient communication or on patients’ treatment goals. This is in line with previous research on primary care which shows that regular meetings in which care providers share knowledge and learn with, from and about each other are regarded by many as being complex, hectic and lacking a clear structure and objective [43–47]. However, when members of a practice team lack sufficient time to plan, assess and evaluate care together, there is a risk of duplication or omission of services, insufficient care coordination and that the synergies from the pooled knowledge and perspectives of team members may not be fully utilised [48–52]. The US Agency for Healthcare Quality and Research has defined effective care coordination approaches to include the creation of a proactive care plan, supporting patients’ self-management goals, case management and linking to community resources [53]. Along similar lines, the Norwegian guidelines for diabetes recommend that patients with diabetes participate in developing their personalized care plan [27]. However, these guidelines do not exemplify the layout or content of these plans. This gap in information is worth noticing, given one of our major findings - namely that none of the practices used care plans to assess patient needs and goals, agreeing on responsibility and sharing information about patient care activities.

Diverse professional backgrounds and care approaches may improve the comprehensiveness of care when assembled into a congruent whole [54,55]. However, collaborative practice requires a shift in the perspective of care providers and the authorities that govern the standardization of professional roles and responsibilities [44,56,57]. Physicians are used to working independently and several GPs in our study admitted to have colleagues in their practice who preferred not to refer patients to cHCPs. They attributed this to disagreement about roles and cHCPs' need for competence. The disagreement between nurses and medical secretaries about each other’s importance in diabetes care may reflect that introduction of new roles in general practice is still only at an early stage.

The GPs confirmed that their consultations were busy, with minimal opportunity for patients to ask questions or receive diabetes education. Previous hospital-based research has demonstrated that involving certified diabetes educators taught in case management principles may lead to improved patient care and reduced hospital readmissions [58]. Enabling patients to feel capable of taking responsibility for their health is a primary goal of diabetes education interventions, as costs and complications associated with diabetes (e.g. end-stage renal disease, blindness and amputations) are largely preventable and related to lifestyle [59]. Based on Norwegian register data and data from electronic medical records, only 9.8% of patients with T1DM and 16% of patients with T2DM achieve combined treatment targets for HbA1c, blood pressure and cholesterol [60,61]. Furthermore, a recent study from general practice found that only 5% of patients with T2DM and coronary heart disease reach the four main treatment targets (no smoking, HbA1c ≤7.0%, SBP ≤135 mmHg, LDL-cholesterol ≤1.8 mmol/l) [62]. GPs have insufficient time to provide such self-management support and often lack general behavioural change skills [63]. We found that nurses and medical secretaries, spending more time with each patient and focusing solely on diabetes, had a professional approach that is better aligned with PCC and therefore may serve an important role in fostering adherence to the self-management regimens in diabetes.

In Norway, there are no official strategies or financial incentives to support general practices in transitioning into team-base care. Reflecting this lack of background information, GPs used terms such as *I delegate*, *they serve me*, *my assistants*, and *I control their work* when they were asked to describe how multi-professional diabetes care was organized in their practice. In contrast, the philosophical underpinnings in literature on collaborative practice is based on ‘we as a team’, shared learning, responsibility and goals [64,65].

Diabetes care was chosen as a case for this study as it is a typical chronic disease for which team-based approaches have been widely applied globally and are recognized as being both beneficial and effective [66–69]. The pursuit of a collective approach in complex cases is recognized as being important to the development of more comprehensive and coherent response to patient needs [35,70]. Thus, the general preference of GPs to not engage cHCPs in the care of complex patient cases (e.g. patients with ‘difficult-to-control’ diabetes or severe multimorbidity) is another key finding in our study. However, despite organizing diabetes care through delegation, participants from all three professional backgrounds referred to their diabetes model as being team-based. We propose that this contradiction relate again, to a lack of attention to team-based care processes in primary care in general and in missing guidelines and regulatory frameworks.

Although collaboration and teamwork are necessary in healthcare, they occur along a continuum [71]. Theoretical literature suggests that successful teams are recognized by the dynamic interaction between team members who adapt interdependently, have a common mission and clear goals, share knowledge and are led by someone who stimulates self-reflection and openness [65,72]. In order to achieve this, members of high performing teams have a clear appreciation of everyone’s roles and tasks and shared care planning runs seamlessly [65]. We suggest that teamwork is not generic and should not be defined simply in terms of content and professional roles. Attention must be paid to the range of healthcare environments within which teamwork is delivered, as well as its external and internal mediators and moderators. Indeed, HCPs working in general practice are under immense pressure to get things done and most often do not have the capacity to introduce changes that are not required, enabled and resourced [73]. Hence, preparing HCPs for collaborative practice requires the development of a framework that is informed by cultural and historical professional traditions, work contexts and accountability mechanisms [44,57,74]. Similarly to our findings, in a qualitative study of Australian general practices, McInnes *et al.* found that GPs and general practice registered nurses had few formal opportunities to discuss long term goals or participate in joint decision-making [6]. Notably, although a lack of professional interaction may hamper the delivery of coordinated care, the GPs in their study indicated that such meetings were a waste of time and also logistically difficult to arrange [6].

Improving healthcare integration and cohesion is high on the political agenda [75,76]. Yet, despite decades of research on professional collaboration, little is known about its direct impact on patient outcomes in primary care [13,77]. There is also a dearth of empirical research on team-based reflection and dynamics [78]. Thus, further research is warranted into inter-professional processes and the effectiveness of different team-based approaches to understand how services can become more coherent, responding to patients’ expectations and needs [2,79].

### Strengths

The purposively selected practices allowed us to generate information about HCP’s experiences of team-based diabetes care that have not been previously reported. The setting in which the interviews took place was non-interventional and participants’ responses directly reflected their actual daily practice. All teams had operated for many years within similar regulatory frameworks, representing urban and rural districts of Norway. The practices offered comparable services in primary care but also had diverse characteristics, allowing our findings to be extended across more than one case. Our comparative approach permitted us to identify similarities and differences among different professional groups in five practices and substantiate the findings across our data.

The analysis was iteratively reviewed by two members of the research team in order to improve the thoroughness of data interpretation. Although several influences examined in our study have been previously addressed in the literature, we bring further nuances to a number of these elements. For example, participating HCPs felt that their approach to diabetes care reflected teamwork, although their practice was more similar to selective delegation and parallel care. We also found that cHCPs responded with flexibility and acceptance when they were delegated partial responsibility instead of requesting participation in the total of care of patients with diabetes.

### Limitations

The practices were purposively sampled and regarded as leading in the specific field of diabetes care. Hence, they are not representative of the average Norwegian general practice. Moreover, their style of collaboration may be affected by human relationships and personalities as much as context, traditions and policies. Readers should also take note of our consideration of medical secretaries and nurses as one group, and that some of the nurses had training as diabetes specialist nurses. We realise that participants represented a heterogenous group and that their various professional backgrounds and experiences may have impacted their responses.

All cHCPs were female with extensive practice experience and all but one GP were male. As discussed above, gender may have impacted the participant’s descriptions of their care approaches and their perception of own role. Nevertheless, studies from related settings in other countries show similar results [80–82], and by providing rich contextual descriptions, we believe our findings could be transferable to comparable settings. Logistical constraints meant it was not possible to carry out a member checking process, which could have further developed the study findings. The main author has been involved in developing the current national clinical guidelines for diabetes. This engagement might have influenced her interpretation of the results. However, as the co-authors in this article have not been involved in developing these guidelines, their involvement in discussing and analysing the empirical data has served to ensure an analytical distance to the guidelines, as well as to reach an agreement in terms of the final themes.

## Conclusion

This study shows that cHCPs, working independently under delegated responsibility, appear to develop their roles by focusing on patient needs for emotional support and having their questions answered , which seem to be given less priority in GP-led consultations. However, by having minimal interaction and not using care plans to align patient care goals, GPs and cHCPs may miss out on important advantages of working in a multi-professional environment. Our findings also indicate that institutional structures such as discriminatory remuneration systems, lack of role descriptions and missing standards for MPC, may hinder the transition to team-based care approaches in Norwegian general practice. Further research is requested in order to understand how gender might affect HCPs’ inclination to provide person-centred care and what elements of MPC contribute to improving patient outcomes.
